# Fine Particulate Matter (PM) Effects on Swine Granulosa and Ovarian Endothelial Cells

**DOI:** 10.3390/ani16010081

**Published:** 2025-12-27

**Authors:** Giuseppina Basini, Roberto Ramoni, Stefano Grolli, Simona Bussolati, Laura Assogna, Francesca Grasselli

**Affiliations:** Dipartimento di Scienze Medico-Veterinarie, Università degli Studi di Parma, Via del Taglio 10, 43126 Parma, Italy; roberto.ramoni@unipr.it (R.R.); stefano.grolli@unipr.it (S.G.); simona.bussolati@unipr.it (S.B.); laura.assogna@studenti.unipr.it (L.A.); francesca.grasselli@unipr.it (F.G.)

**Keywords:** ovary, granulosa cells, endothelial cells, ROS, VEGF, estrogen, progesterone

## Abstract

We studied fine particulate matter (PM) effects on porcine granulosa, a model of endocrine reproductive cells, and on ovarian endothelial cells. PM produced a significant decrease (*p* < 0.05) in metabolic activity. PM reduced endothelial cell proliferation (*p* < 0.001). As for redox status, superoxide anion (O_2_^−^) production by granulosa was reduced (*p* < 0.05) by PM, while it was increased in endothelial cells (*p* < 0.001). PM increased (*p* < 0.001) nitric oxide (NO) levels in granulosa, while in endothelial cells, it displayed a biphasic effect (*p* < 0.05) as well as for superoxide dismutase activity (SOD) in granulosa cells (*p* < 0.001). In endothelial cells, PM increased (*p* < 0.001) SOD. A reduction in Ferric Reducing Ability of Plasma (FRAP) (*p* < 0.01) was observed. In granulosa, PM did not induce oxidative damage to DNA, while in endothelial cells, it determined a reduction (*p* < 0.05). PM significantly inhibited steroidogenesis (*p* < 0.05). The accumulation of autophagic vacuoles and Vascular Endothelial Growth Factor (VEGF) production was increased (*p* < 0.05) by PM. The data obtained likely demonstrate that PM induces critical effects on ovarian cells.

## Simple Summary

Environmental pollution is an increasing threat to the health of both humans and animals. Fine particulate matter, a component of air pollution, is known to penetrate deep into the body, but its specific effects on fertility are not yet fully understood. This study aimed to determine how these particles affect the ovary. We analyzed the effects on granulosa cells (which surround and support the egg) and endothelial cells (which form the blood supply). The results demonstrated that exposure to pollution negatively affects both cell types, impairing their growth and triggering cellular distress. These results suggest that the ovary is particularly sensitive to airborne pollutants. Understanding these effects is crucial for animal welfare and veterinary medicine, as well as human health, as it links poor air quality to compromised reproductive potential. These findings support the case for severe environmental monitoring to safeguard fertility across species.

## 1. Introduction

The rapid industrial and urban development has exacerbated air pollution, which represents a significant health concern in many developed countries [1]. Air pollutants include a complex mixture of gases and particulate matter (PM). Based on aerodynamic diameter, particulate matter (PM) is typically classified into PM10 (<10 μm), PM2.5 (<2.5 μm), and ultrafine particles (UFPs, 1–100 nm) [2]. PM contains a variety of toxic components, with water-soluble extracts being mainly composed of highly soluble and bioavailable metals, as well as polycyclic aromatic hydrocarbons (PAHs) that feature a small number of rings [3]. PM2.5 is a primary type of particulate matter pollution. Due to its small particle size, PM2.5 can be present in the air for extended periods, easily entering the lungs during respiration. Once in the lungs, PM2.5 can enter blood vessels reaching by means of the bloodstream various organs and systems, potentially causing significant health damage [4].

Based on the available evidence, a multi-step hypothesis for the delivery and sequestration of PM2.5 in the ovary can be formulated:

1. Inhaled PM2.5 particles, along with their adsorbed toxic chemical payload, are deposited in the lung alveoli [5].
2. A fraction of these particles and/or their soluble components translocate across the air-blood barrier, entering the systemic circulation [5]. Concurrently, a pulmonary inflammatory response releases cytokines and other mediators into the blood [6,7,8].
3. These circulating particles and soluble toxicants are transported throughout the body and are delivered to the ovary via its extensive arterial blood supply (the ovarian artery) [7,9].
4. Within the ovarian microenvironment, particles may extravasate from the capillaries and become sequestered in the ovarian stroma or interstitial tissue. From here, they can directly interact with the cells of the follicular unit—including theca cells, granulosa cells, and the oocyte—as well as the endothelial cells of the ovarian vasculature [9].
5. This direct, localized exposure to a complex mixture of toxicants, superimposed upon a state of systemic inflammation induced by the pulmonary response [10], initiates the cascade of cellular damage, including oxidative stress, inflammation, disrupted cell replication, and altered endocrine function.

Therefore, ovarian granulosa and endothelial cells could be affected by a double insult: direct damage from translocated particles and their chemical constituents, and indirect damage from a systemic inflammatory and oxidative state orchestrated by the initial pulmonary injury.

Infertility represents a pressing public health issue as global fertility rates are steadily declining. According to studies, the global total fertility rate (TFR) has dropped steadily, with notable decreases observed between 1990 and 2019. Key drivers of this decline include reduced fertility desires due to socioeconomic changes, delays in childbearing, and increased infertility rates [11]. Biological, environmental, and lifestyle factors significantly contribute to infertility [12]. Recently, air pollution has gained attention as a critical environmental factor disrupting reproductive health [13]. Pollutants such as particulate matter (PM2.5) are suspected to impact fertility in both males and females, even if molecular mechanisms are unclear at present [14]. Chen et al. [15] previously demonstrated that PM2.5 exposure can seriously impair ovarian function, but they did not clarify the underlying mechanisms. It is well known that the ovarian follicle, which houses the developing oocyte, is not directly bathed in systemic blood. Instead, it is protected by the blood-follicular barrier, a multi-layered physiological barrier that selectively regulates the passage of substances from the bloodstream into the follicular fluid surrounding the oocyte and granulosa cells. However, Zhang et al. [16] documented ovarian damage in mice, evidenced by inflammatory cell infiltration and follicular atresia, following a 5-month exposure to PM2.5 via tracheal drip. They found that Activating Transcription Factor 3 (ATF3) is involved in PM2.5-induced inflammation and apoptosis in ovarian granulosa cells. Nevertheless, the precise mechanism of PM-induced damage to ovarian granulosa cells remains unclear.

Therefore, the present research has focused on the effects of diesel-derived microparticulate matter (PM) on granulosa and endothelial cells isolated from swine ovaries. Traditionally, most reproductive toxicology studies have been carried out in vivo. The need to reduce animal use in toxicological testing has led to the development of alternative cell culture techniques. These in vitro systems represent a significant advancement in the field of toxicology, offering the potential to complement or even replace conventional in vivo models. Compared to traditional methods, they provide ethical, cost-effective, and reproducible alternatives for evaluating reproductive toxicity [17]. The swine species has been chosen since it is considered a valid model for translational medicine [18,19,20]. Porcine biomedical models have significantly contributed to advancing our understanding of key reproductive processes in humans, including puberty, fertilization, pregnancy, and reproductive pathologies (reviewed in Mordhorst and Prather [21]). The reproductive similarities between pigs and humans that support the use of pigs in translational research have been described in the paper by Lunney et al. [22]. These features make swine useful for reproductive toxicological research, even if species-specific differences should be considered as regards some aspects of reproductive processes. Cultured porcine granulosa cells, used as a validated model of endocrine cells [23,24,25], were treated with PM (5, 10, and 20 µg/mL) [23,24,25,26]. The same PM concentrations were used on endothelial cells collected from the porcine corpus luteum, a transitory gland whose development is strictly dependent on angiogenesis. It is well known that CL is essential to produce progesterone, a hormone involved in the maintenance of pregnancy. The effects of PM on both cell types have been focused on cell proliferation evaluated by Bromo-deoxy-Uridine incorporation, metabolic activity, by means of ATP production, and redox status, measuring generation of superoxide anion through the WST test. We also evaluated the non-enzymatic antioxidant power through the Ferric Reducing Ability of Plasma (FRAP) test, superoxide dismutase (SOD) and 8-hydroxydeoxyguanosine (8-OHdG) activity, as an indicator of oxidative damage to DNA, as well as nitric oxide (NO) production measured using the Griess test. Moreover, the research was focused on the evaluation of a possible impact on granulosa cells’ endocrine activity, measuring the production of sexual steroids, estrogen, and progesterone, through ELISA immunoassays. Furthermore, the autophagy process has been considered using an ELISA assay. Instead, limited to endothelial cells collected from the corpus luteum, the effect on Vascular Endothelial Growth Factor (VEGF), essential for angiogenesis, has been considered. Therefore, the present study aimed to verify the potential alteration of ovarian homeostasis and disruption of endocrine function induced by PM that could critically impact fertility in the sow, which is also a valuable translational model for human reproduction impairment.

## 2. Materials and Methods

All reagents used in this study were obtained from Merck (Darmstadt, Germany) unless otherwise specified, while plastic material was from Sarstedt AG & Co. (Numbrecht, Germany).

### 2.1. Isolation and Culture of Swine Granulosa Cells and Endothelial Cells from Swine Corpus Luteum

At least five collections of ovaries were realized at a local abattoir from 40 Large White cross-bred gilts, parity = 0, at a local slaughterhouse (Macello Pini Italia s.r.l., Castelverde, CR, Italy). Based on previous observations [27,28,29,30,31], the stage of the oestrous cycle was determined by evaluating ovarian morphology. The ovaries were immersed in sterile phosphate-buffered saline (PBS, 4 °C) containing 500 IU/mL penicillin, 500 µg/mL streptomycin, and amphotericin B (3.5 µg/mL) and transferred to the lab. Ethanol 70% was chosen for cleaning, followed by washing in sterile PBS [32,33,34]. Ovarian follicles were classified based on morphological criteria [35]. Granulosa cells were collected under sterile conditions from healthy follicles larger than 5 mm using a medium added with 50 IU/mL heparin [36,37,38]. Mural granulosa cells were retrieved by gently scraping the follicle wall with a needle. Cell purity consistently exceeded 90%. Cells were cultured in a previously validated DMEM/Ham’s F12 culture medium added with bovine serum albumin (BSA 1 mg/mL), penicillin (100 IU/mL), streptomycin (100 µg/mL), amphotericin B (2.5 µg/mL), selenium (5 ng/mL) and transferrin (5 µg/mL) (CM) [39]. The use of this medium is essential to prevent the luteinization of granulosa cells, which can maintain their differentiation.

Isolation of endothelial cells from ovaries was carried out as previously described [40]. A more detailed description of the modified isolation protocol and cell characterization is included in our earlier study [41]. Briefly, working under sterile conditions, portions of parenchyma containing clearly visible blood vessels were selected from corpora lutea, placed in a drop of medium on a sterile wooden support, coarsely fragmented with a scalpel blade, and then more finely shredded with a multilama knife. The resulting homogeneous pulp was centrifuged (500× *g* for 10 min); the pellet was resuspended and, thereafter, three washes were performed (500× *g* for 10 min). The final cell suspension was initially filtered through sterile gauze (150 mesh) and subsequently through a 70 µm filter (BD Falcon, Bedford Bioscience, Bedford, MA, USA). To remove red blood cells, the suspension was treated with 0.17 M NH_4_Cl for 1 min and centrifuged at 500× *g* for 10 min. Thereafter, 500 µL of the cell suspension was seeded into 25 cm^2^ flasks containing 5 mL of EBM-2 supplemented with EGM-2 (Clonetics, Lonza, Walkersville, MD, USA). A change of culture medium was performed every 48 h to discard non-adherent cells. Both granulosa and endothelial cells were incubated for 48 h at 37 °C in an environment of 5% CO_2_ and 95% humidified air. The incubation was conducted with or without fine particulate matter (PM) (SRM^®^ 1650b; NIST, CAS 1333-86-4, Sigma-Aldrich, Merck, Darmstadt, Germany) resuspended in medium at concentrations of 5, 10, and 20 μg/mL. This range of concentrations has already been explored in both human and animal (mouse, porcine) endothelial cells [26,42,43,44]. Regarding granulosa cells, a recent study by Zhang et al. [16] explored the possible cytotoxic role of PM on the human granulosa cell line KGN at concentrations ranging from 50 to 150 μg/mL. In recent years, several studies have attempted to clarify the dispersion and sedimentation processes of PM suspended in serum or protein-supplemented culture media. Under these conditions, PM rapidly forms stable “secondary agglomerates” mediated by the so-called “protein corona” [45], which prevents uncontrolled precipitation while facilitating gravitational sedimentation. Application of sedimentation models to these aggregates demonstrates that their sedimentation rate is sufficient to deliver nearly 100% of the nominal mass to the cell monolayer within the first 24 h of exposure [46]. Consequently, for the 48-h duration of this study, we think that the nominal concentration used serves as an accurate proxy for the effective contact concentration.

### 2.2. Cell Proliferation

Granulosa and endothelial cell proliferation were assessed using the Bromodeoxyuridine (BrdU) incorporation assay (Roche Diagnostics, Mannheim, Germany). Briefly, after seeding 10^4^ granulosa cells per well and 1.5 × 10^3^ endothelial cells per 200 µL of medium into 96-well plates, a 24 h incubation at 37 °C in an environment with 5% CO_2_ and 95% humidified air was carried out. Then, after medium removal, we proceeded to a 48-h treatment with 5, 10, and 20 µg/mL of PM, as previously described. We then added 20 µL BrdU label to each well and incubated overnight. Centrifugation was carried out at 400× *g* for 10 min, the medium was discarded, and 200 µL of FixDenat Solution was added to enhance antibody detection of incorporated BrdU. A conjugated anti-BrdU antibody was then applied, and immune complex detection was performed through a subsequent substrate reaction. A Victor Nivo spectrophotometer (Perkin Elmer, Groningen, The Netherlands) was employed for the measurement of the product absorbance at 450 nm (Perkin Elmer, Groningen, The Netherlands) [47].

### 2.3. Cell Metabolic Activity

After seeding into 96-well plates, 2 × 10^5^ viable granulosa cells and 5 × 10^4^ endothelial cells per 200 µL medium were treated with PM for 48 h, as previously reported. A bioluminescent assay (ATP-lite 1-step; Perkin Elmer, Groningen, The Netherlands) was used to quantify intracellular ATP levels, serving as a marker of cell viability. The reaction of ATP with D-luciferin, catalyzed by luciferase, produces light whose intensity is directly proportional to ATP concentration. Briefly, 100 µL of cell suspension was mixed with 100 µL of substrate solution, and a Victor Nivo spectrophotometer was employed for luminescence quantification [48].

### 2.4. Cell Redox Status

#### 2.4.1. Non-Enzymatic Scavenging Activity

The Ferric Reducing Ability of Plasma (FRAP) assay was employed to evaluate the antioxidant capacity of biological samples by measuring their ability to reduce ferric-tripyridyltriazine (Fe^3+^-TPTZ) to ferrous (Fe^2+^-TPTZ). Granulosa (2 × 10^5^ cells/well) and endothelial (5 × 10^4^ viable cells/well) cells were seeded in 96-well plates and treated with PM for 48 h under standard incubation conditions (37 °C, 5% CO_2_, and 95% humidified air), as previously described. After incubation, centrifugation was carried out as described above, supernatants were discarded, and immersion in an ice bath for 30 min using a cold Triton 0.5% + PMSF in PBS solution (200 µL/well) resulted in cell lysis. To assess the reducing ability, 40 µL of cell lysate was added to the Fe^3+^-TPTZ reagent and incubated for 30 min at 37 °C. The reduction reaction produced a blue colour, which was measured at 595 nm using a Victor Reader spectrophotometer. The antioxidant capacity was quantified by comparing absorbance values to a standard curve generated from FeSO_4_-7H_2_O [47].

#### 2.4.2. Enzymatic Scavenging Activity: Superoxide Dismutase (SOD)

Superoxide dismutase (SOD) activity was determined using the SOD Assay Kit (Sigma Chemical Co., Ltd., St. Louis, MO, USA) according to the manufacturer’s instructions. Granulosa cells (2 × 10^5^ per well) and endothelial cells (5 × 10^4^ per well) were cultured in 96-well plates (Sarstedt, Nümbrecht, Germany) and treated with PM for 48 h, as previously described. Following incubation and cell centrifugation, the supernatants were removed, and cell lysis was performed using the previously described method. Cell lysates were analyzed without dilution, and a standard curve of SOD activity (0.156–20 U/mL) was prepared. The assay measured formazan formation, which results from the reaction between a tetrazolium salt and superoxide anion (O_2_^−^), generated by an exogenous xanthine oxidase reaction. The endogenous SOD activity was indirectly evaluated by determining the remaining O_2_^−^ levels. Absorbance was quantified by means of a Victor Nivo spectrophotometer at 450 nm, with a reference wavelength of 620 nm [25].

### 2.5. Nitric Oxide (NO) Production

We seeded granulosa cells (2 × 10^5^ cells/well) and endothelial cells (5 × 10^4^ cells/well) in 96-well plates and subjected them to a 48-h incubation under standard conditions with PM as previously described. Following incubation, cell centrifugation was carried out, and the supernatants were collected. Nitrite levels, an indicator of NO production, were quantified using the Griess test, which measures the accumulation of nitrite in cell culture supernatants [34,37,49].

### 2.6. Superoxide (O_*2*_^−^) Production

The production of superoxide anion (O_2_^−^) by cultured cells was assessed using the Cell-Proliferation Reagent WST-1 test (Roche Diagnostics, Indianapolis, IN, USA), as described in previous studies [33,50]. We cultured granulosa cells (2 × 10^5^ cells/well) and endothelial cells (5 × 10^3^ cells/well) in 96-well plates for 48 h under standard conditions in the presence of PM at the previously specified concentrations. During the last 4 h of incubation, we added 20 µL of WST-1 reagent to each well. The absorbance was then quantified at 450 nm, with a reference wavelength of 620 nm, using a Victor Nivo spectrophotometer [48].

### 2.7. 8-Hydroxydeoxyguanosine (8-OHdG)

8-Hydroxydeoxyguanosine (8-OHdG) is a modified DNA base resulting from interactions with hydroxyl radicals, which are byproducts and intermediates of aerobic metabolism. Its levels increase significantly under oxidative stress, making it a valuable biomarker for oxidative DNA damage [51]. After seeding, granulosa cells (2 × 10^5^ cells/well) and endothelial cells (5 × 10^3^ cells/well) were subjected to a 48-h treatment with PM at the previously specified concentrations. To quantify 8-OHdG levels, a competitive ELISA assay was performed using the 8-OHdG ELISA Kit (ab201734; Abcam, Cambridge, UK). This kit detects both free 8-OHdG and DNA-incorporated 8-OHdG. The ELISA assay was conducted on an 8-hydroxy-2-deoxyguanosine-coated plate, utilizing an HRP-conjugated antibody for detection. Conditioned media samples (25 µL for granulosa or 50 µL for endothelial cells) were loaded into the 96-well plate provided by the kit and added with BSA. Absorbance has been recorded by Victor Nivo microplate reader at 450 nm. The assay range is 0.94–60 ng/mL. Assay sensitivity is 0.59 ng/mL.

### 2.8. Granulosa Cell Steroidogenesis

Ten thousand viable cells were cultured in 96-well plates with 200 µL of medium supplemented with 28 ng/mL androstenedione [52] with or without PM as previously described. After storage at −20 °C, we quantified progesterone (P4) and estradiol 17β (E2) levels in collected media by means of Estradiol and Progesterone ELISA kits (Dia.Metra s.r.l, Spello, PG, Italy). As for the E2 assay, sensitivity is 8.6 pg/mL, and the intra-assay CV is <9%; the P4 assay sensitivity is 0.05 ng/mL, and the intra-assay CV is <4%. Victor Nivo reader (Perkin Elmer, Groningen, The Netherlands) [53] was used to read absorbance at 450 nm against a reference wavelength of 620–630 nm.

### 2.9. Autophagy in Granulosa Cells

Autophagy is an evolutionarily conserved process by which cytoplasmic components and organelles are degraded via lysosomes; therefore, it results in a regulated turnover of cellular constituents during development or in response to stress. Autophagy plays a positive role for the cell because it allows survival in the absence of nutrients, development, and elimination of intracellular components. In 96-well plates, 2 × 10^5^ cells/well/200 µL medium were plated, then treated with PM for 48 h. This analysis has been performed on cell lysates, collected after a five-minute centrifugation performed at 400× *g* at room temperature. “Autophagy assay kit” has been employed (Abnova Corporation, Taoyuan, Taiwan, No. KA4076). In each well, 50 µL of lysate was added with 50 µL of the Autophagy Blue working solution, prepared following the instructions of the kit, mixing well the components in 10 µL of Stain Buffer. The plate is incubated at 37 °C for 45 min and, using the Victor Nivo microplate reader, a fluorescence reading is performed with an excitation length of 355/40 nm and an emission length of 530/30 nm.

### 2.10. Endothelial Cell Vascular Endothelial Growth Factor (VEGF) Production

VEGF-A levels in spent media were quantified by an ELISA test (Quantikine, R&D System, Minneapolis, MI, USA), validated for porcine VEGF [54,55]. The assay sensitivity was 0.23 pmol/L. The intra- and inter-assay coefficient of variation (CV) was <7%. After culturing, 5 × 10^4^ cells in 200 µL of medium for 24 h, the media were discarded, and the cells were subjected to a 48-h treatment with PM as described.

### 2.11. Statistical Analysis

The experiments were repeated at least five times with six replicates for each treatment. Data are presented as mean ± SEM (standard error of mean). Statistical difference was calculated by One-Way ANOVA using Statgraphics software (version 5 PLUS, STC Inc., Rockville, MD, USA). In the presence of a significant difference (*p* < 0.05), the means were subjected to the Scheffè’s F test for multiple comparisons.

## 3. Results

### 3.1. Granulosa Cell Growth

**Figure 1:**
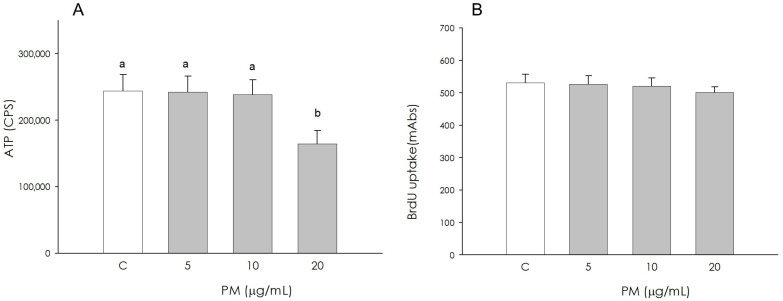
Effect of the treatment with fine particulate matter (PM 5, 10, and 20 µg/mL) for 48 h on swine granulosa cell metabolic activity using ATP content assay test (**A**) and proliferation using 5-bromo-2’-deoxyuridine (BrdU) incorporation assay test (**B**). Data, expressed as counts per second (CPS) in panel (**A**) and as milliabsorbance units (mAbs) in panel (**B**), represent the mean ± SEM of six replicates/treatment repeated in five different experiments. Different letters on the bars indicate a significant difference (*p* < 0.05) among treatments as calculated by ANOVA and Scheffè’s F test.

Granulosa cell metabolic activity by quantifying ATP production was not influenced by PM at 5 and 10 μg/mL, while it resulted in a significant reduction (*p* < 0.05) by the treatment at the highest concentration (20 μg/mL) (Figure 1A). Granulosa cell proliferation, assessed by the incorporation of BrdU into newly synthesized DNA, was not affected by the PM treatment at 5, 10 and 20 μg/mL (Figure 1B).

### 3.2. Granulosa Cell Steroidogenesis

**Figure 2:**
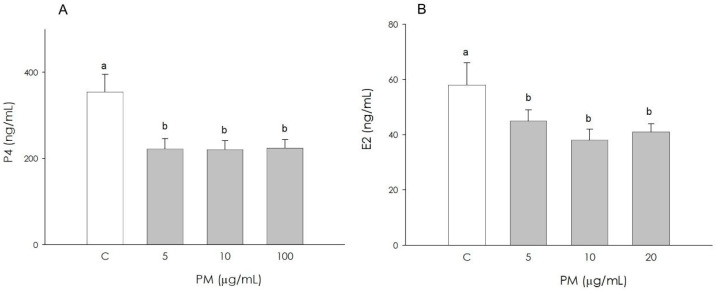
Effect of the treatment with fine particulate matter (PM 5, 10, and 20 µg/mL) for 48 h on swine granulosa cell progesterone (**A**) and estradiol 17β (**B**) production, using ELISA assays. Data, expressed as ng/mL, represent the mean ± SEM of six replicates/treatment repeated in five different experiments. Different letters on the bars indicate a significant difference (*p* < 0.05) among treatments as calculated by ANOVA and Scheffè’s F test.

Treatment with PM, at all the examined concentrations, negatively influences the levels of both progesterone (P4) (*p* < 0.001) and estradiol 17β (E2) (*p* < 0.05) in granulosa cells (Figure 2A,B).

### 3.3. Granulosa Cell Redox Status

**Figure 3:**
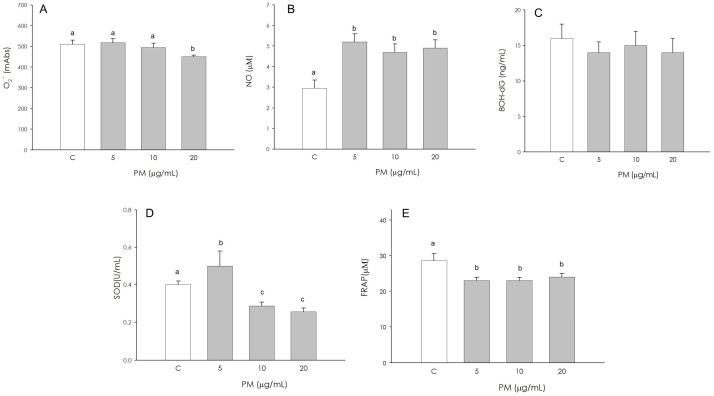
Effect of the treatment with fine particulate matter (PM 5, 10 and 20 µg/mL) for 48 h on swine granulosa cells superoxide anion (O_2_^−^) generation using colorimetric assay (**A**), nitric oxide (NO) production using Griess Assay (**B**), 8-hydroxydeoxyguanosine (8OH-dG) using colorimetric assay (**C**), superoxide dismutase activity using SOD assay (**D**) and non-enzymatic scavenging activity using the FRAP assay (**E**). Data, expressed as milliAbs units (panel (**A**)), as µM (panel (**B**) and (**E**)), as ng/mL (panel (**C**)), and as U/mL (panel (**D**)), represent the mean ± SEM of six replicates/treatment repeated in five different experiments. Different letters on the bars indicate a significant difference (*p* < 0.05) among treatments as calculated by ANOVA and Scheffè’s F test.

Superoxide anion (O_2_^−^) production was not influenced by the treatments with 5 and 10 µg/mL, while at the higher concentration (20 µg/mL), a significant decrease (*p* < 0.05) of O_2_^−^ production was highlighted (Figure 3A). Exposure to PM caused a significant increase (*p* < 0.001) in nitric oxide (NO) production at 5, 10, and 20 μg/mL, with no significant difference among them (*p* < 0.001) (Figure 3B). On the contrary, the production of 8-hydroxydeoxyguanosine (8OH-dG) by granulosa cells was not affected by PM treatment (Figure 3C). An increase (*p* < 0.001) of superoxide dismutase (SOD) activity was observed in granulosa cells treated with 5 µg/mL. A decrease (*p* < 0.001) in SOD activity was observed at the intermediate (10 µg/mL) and highest (20 µg/mL) concentrations (Figure 3D). Non-enzymatic antioxidant activity was significantly inhibited (*p* < 0.01) by exposure to 5, 10, 20 µg/mL PM. The antioxidant power in baseline conditions was visibly higher than that of the treated groups (Figure 3E).

### 3.4. Granulosa Cell Autophagy

**Figure 4:**
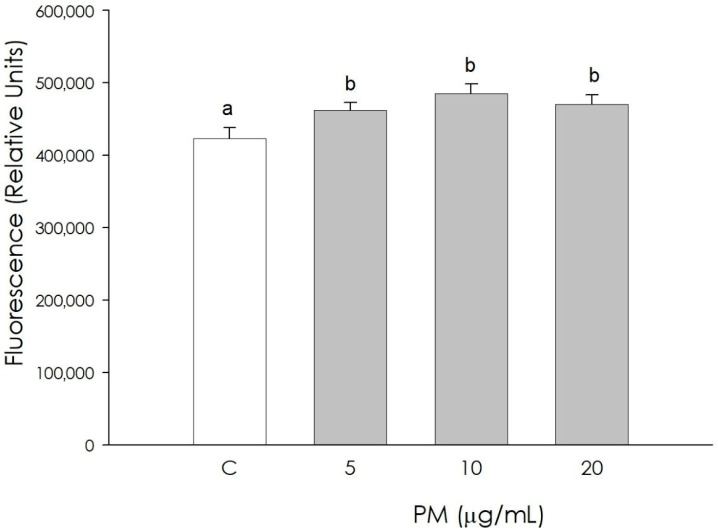
Effect of the treatment with fine particulate matter (PM 5, 10, and 20 µg/mL) for 48 h on swine granulosa cells autophagy using fluorimetric assay. Data, expressed as fluorescence relative units, represent the mean ± SEM of six replicates/treatment repeated in five different experiments. Different letters on the bars indicate a significant difference (*p* < 0.05) among treatments as calculated by ANOVA and Scheffè’s F test.

The autophagy phenomenon in granulosa cells was increased (*p* < 0.05), following treatment with PM at all concentrations used (5, 10, 20 µg/mL) (Figure 4).

### 3.5. Endothelial Cell Growth

**Figure 5:**
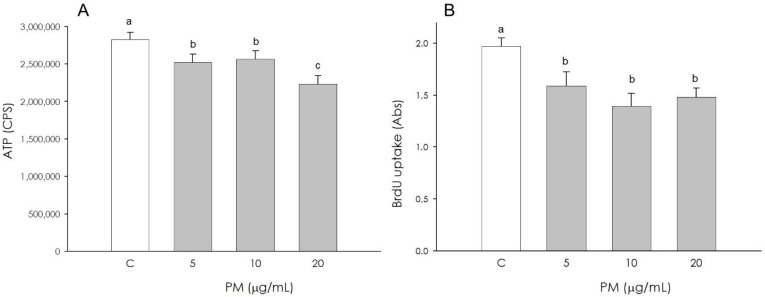
Effect of the treatment with fine particulate matter (PM 5, 10, and 20 µg/mL) for 48 h on swine endothelial cell metabolic activity using ATP content assay test (**A**) and proliferation using 5-bromo-2’-deoxyuridine (BrdU) incorporation assay test (**B**). Data, expressed as counts per second (CPS) in panel (**A**) and as milliabsorbance units (mAbs) in panel (**B**), represent the mean ± SEM of six replicates/treatment repeated in five different experiments. Different letters on the bars indicate a significant difference (*p* < 0.001) among treatments as calculated by ANOVA and Scheffè’s F test.

Metabolic activity, assessed by quantifying ATP production, was negatively influenced by the presence of PM treatment at all concentrations used (5, 10, 20 µg/mL) (*p* < 0.001); in particular, a marked effect (*p* < 0.001) was observed in the presence of 20 μg/mL (Figure 5A). Cell proliferation, assessed by BrdU incorporation into newly synthesized DNA, was decreased (*p* < 0.001) in cells treated with PM in the presence of all concentrations used (5, 10, 20 µg/mL) (Figure 5B).

### 3.6. Endothelial Cell VEGF Production

**Figure 6:**
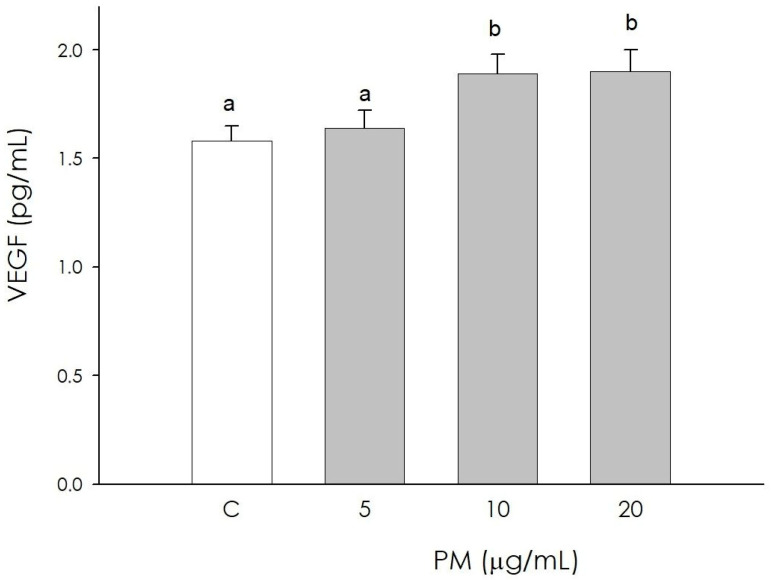
Effect of the treatment with fine particulate matter (PM 5, 10, and 20 µg/mL) for 48 h on swine endothelial cells’ VEGF production using ELISA assay. Data, expressed as pg/mL, represent the mean ± SEM of six replicates/treatment repeated in five different experiments. Different letters on the bars indicate a significant difference (*p* < 0.05) among treatments as calculated by ANOVA and Scheffè’s F test.

Angiogenic activity was assessed by measuring VEGF in corpus luteum endothelial cells. At the lowest PM concentration (5 µg/mL), no significant difference was observed in the production of this growth factor compared to the control; treatments at higher concentrations (10 and 20 µg/mL), however, determined a significant increase (*p* < 0.05) (Figure 6).

### 3.7. Endothelial Cell Redox Status

**Figure 7:**
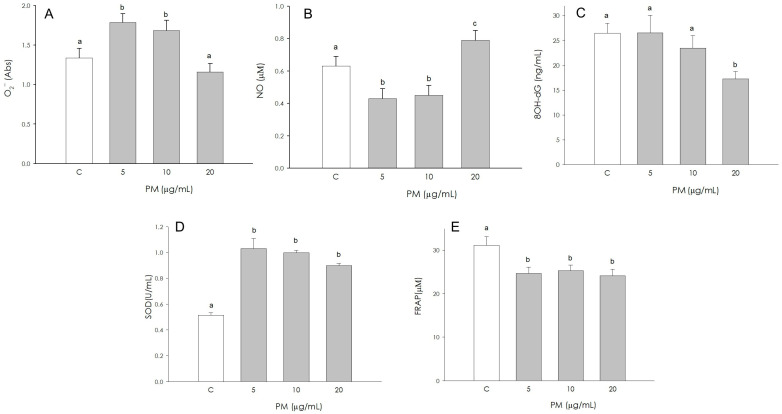
Effect of the treatment with fine particulate matter (PM 5, 10 and 20 µg/mL) for 48 h on swine endothelial cells superoxide anion (O_2_^−^) generation using colorimetric assay (**A**), nitric oxide (NO) production using Griess Assay (**B**), 8-hydroxydeoxyguanosine (8OH-dG) using colorimetric assay (**C**), superoxide dismutase activity using SOD assay (**D**) and non-enzymatic scavenging activity using the FRAP assay (**E**). Data, expressed as milliAbs units (panel (**A**)), as µM (panel (**B**) and (**E**)), as ng/mL (panel (**C**)), and as U/mL (panel (**D**)), represent the mean ± SEM of six replicates/treatment repeated in five different experiments. Different letters on the bars indicate a significant difference (*p* < 0.05) among treatments as calculated by ANOVA and Scheffè’s F test.

O_2_^−^ production was significantly stimulated (*p* < 0.001) by 5 and 10 µg/mL PM; however, the treatment at a higher concentration (20 µg/mL) was ineffective compared to the control (Figure 7A). NO production resulted in a significant reduction (*p* < 0.05) by 5 and 10 µg/mL PM, while it appeared significantly increased (*p* < 0.05) at 20 µg/mL (Figure 7B). The production of 8-OHdG was not affected by treatment with 5 µg/mL and 10 µg/mL PM; the treatment with a higher concentration (20 µg/mL) determined a reduction (*p* < 0.05) in the production of 8-OHdG (Figure 7C). PM significantly stimulated (*p* < 0.001) SOD activity in corpus luteum endothelial cells at all concentrations tested (5, 10, 20 µg/mL) (Figure 7D). Non-enzymatic antioxidant activity was negatively influenced (*p* < 0.001) by PM treatment at all concentrations used (5, 10, 20 µg/mL) (Figure 7E).

## 4. Discussion

Air pollution is a serious environmental emergency that has been classified as a major cause of mortality and disease in Europe by the European Environment Agency. Among the various environmental pollutants, particulate matter (PM) represents a serious threat. PM10 (less than 10 μm aerodynamic diameter) and PM2.5 (less than 2.5 μm) constitute the inhalable fraction of PM.

Exposure to atmospheric PM has been shown to reduce reproductive capacity, delay ovarian development, and cause infertility in several animal species [56,57]. However, despite these critical issues, the effects of PM on the reproductive system and fertility of female mammals remain incompletely known. Therefore, we evaluated the effects of PM (5, 10, and 20 µg/mL) on primary cultures of sow granulosa cells and corpus luteum endothelial cells.

The comparison between in vitro concentrations (µg/mL) and ambient air concentrations (µg/m^3^) often suggests a disparity, leading to doubts about the physiological relevance of laboratory findings. The organisms are not simple inert containers; cells actively interact with and internalize particulate matter. Consequently, the local concentrations of PM within subcellular compartments, such as phagosomes in macrophages, or at the immediate surface of epithelial cells, can be significantly higher than the average concentration found in the surrounding extracellular fluid. It has been demonstrated that alveolar macrophages are highly active phagocytic cells that engulf inhaled particles [58]. It should be noted that granulosa cells can exhibit some macrophage-like functions, particularly in the context of the ovarian environment [59]. Granulosa cells can mimic some macrophage functions, such as phagocytosis (engulfing and digesting cellular debris). These aspects deserve attention and further investigation.

Our study was conducted using pigs reared in northern Italy, the Po Valley, and represents a robust translational model for investigating PM toxicity. Sharing the same areological basin as the local human population, these animals undergo chronic exposure to analogous pollutant profiles, including critical secondary aerosols. The validity of this model is substantiated by the high anatomical and physiological homology between the porcine and human species [60]. Furthermore, the husbandry conditions and sedentary nature of these make the swine an ideal biological sentinel for assessing the systemic health implications of environmental pollution.

In our study, swine granulosa cells exposed to a 48 h treatment with 5, 10, and 20 µg/mL did not show a significant alteration in cell proliferation. Currently, since data in the literature are lacking on this aspect, future studies could be addressed to study the possible effects of different PM concentrations. Endothelial cells derived from the corpus luteum displayed a different proliferative response, since all PM treatments inhibited cell proliferation. These results agree well with those obtained by Okeson et al. [61], who reported a growth rate decrease greater than 40% in lung epithelial cells induced by 12.5 μg/mL PM compared to controls.

PM air exposure can result in oxidative stress due to its physical and chemical characteristics. The effects of PM on cell function can generate ROS, which are reactive metabolites that can influence cellular functions.

Our data show that PM treatment at 5 and 10 µg/mL did not affect O_2_^−^ levels in granulosa cells, while the treatment at higher concentrations caused a significant decrease. These results are difficult to explain, mostly because the in vitro effects of PM on the production of O_2_^−^ are unknown at present. Guo et al. [62] reported that exposure to PM2.5 increased ROS levels in mouse oocytes and consequently induced their degeneration. Liu et al. [9] showed that in Caenorhabditis elegans acutely exposed to PM2.5, a significantly increased ROS production was observed.

Differently, in endothelial cells from the corpus luteum, the production of this ROS was significantly stimulated by 5 and 10 µg/mL PM, while the treatment at 20 µg/mL is ineffective. Rui et al. [63] documented that exposing a human umbilical vein cell line to PM2.5 induces an increase in ROS with a consequent decrease in cell viability. Wang et al. [64] demonstrated that PM- induced ROS production in human lung endothelium disrupts the endothelial cell barrier through the MAP-kinase and HSP27-dependent pathways.

Our results show that PM does not increase 8-OHdG generation in granulosa cells, suggesting that the concentrations tested do not exert significant oxidative harmful effects on DNA in our cell model. Results from in vivo studies are mixed: Lu et al. [65] highlighted that some substances present in PM, such as PAHs and heavy metals, result in oxidative damage to DNA in somatic cells, increasing the 8-OHdG content in the urine of humans who were frequently exposed to these substances. On the other hand, Zhang et al. [66] exposed male rats to PM2.5, demonstrating that 8-OHdG levels in the testes increased only with the highest dose (16.2 mg/kg), while at the two lowest concentrations (1.8 and 5.4 mg/kg), no differences were detected compared to the control.

Free radicals are highly reactive molecules that can damage lipids, proteins, and DNA through oxidative processes. Enzymatic and non-enzymatic antioxidant cell systems are strictly involved in the detoxification of these reactive molecules.

SOD is a key enzyme in the maintenance of cell redox homeostasis thanks to its protective function against oxidative damage. In granulosa cells exposed to the highest levels of PM (10, 20 µg/mL), we observed a decrease in this enzyme, possibly contributing to oxidative stress resulting in several cellular dysfunctions. Moreover, in granulosa cells treated with PM, a decrease in non-enzymatic antioxidant activity was also observed. Since these antioxidants work synergistically to protect cells from oxidative damage, the impairment of redox homeostasis could result in an altered functioning of reproductive processes [67]. ROS and SOD are essential in regulating the correct functionality of the corpus luteum. Increased SOD expression improves the ability to eliminate ROS, prolonging corpus luteum function and consequently the secretion of progesterone [68]. On the contrary, if SOD activity is impaired, a ROS-induced apoptosis of luteinized granulosa cells occurs with consequent regression of the corpus luteum. Therefore, it is evident that SOD and ROS play a crucial role in ovarian physiology, so that a disturbance of their levels caused by PM could disrupt the correct course of the ovarian cycle.

Since the integrity and correct functioning of the vasculature are essential for ensuring ovarian physiology, its impairment could disrupt reproductive function. Also, PM-treated endothelial cells from the corpus luteum showed a reduction in SOD enzymatic activity and non-enzymatic antioxidant power at all concentrations examined. Long et al. [69] exposed human umbilical vein endothelial cells to PM2.5, highlighting that an increase of its concentration from 0 to 100 μg/mL was associated with an inhibition of SOD activity. A decrease in SOD activity was also observed by Kouassi et al. [70] in HaCaT keratinocytes and by Chirino et al. [71], in human lung epithelial cells (A549 cell line), exposed to microparticulate matter. At the same time, ROS levels increased following PM2.5 exposure in the in vitro experiments.

Autophagy is a cellular phenomenon aimed at the degradation or recycling of damaged or no longer necessary cellular components. In the present research, we studied PM influence on the autophagic process. The results obtained show an increase in the accumulation of autophagic vacuoles in granulosa cells following treatment with PM at all concentrations examined. Currently, there is no information in the literature on the effect of PM on the autophagic process of granulosa cells in vitro. This result could be related to the impairment of redox homeostasis due to an alteration of ROS, SOD, and non-enzymatic antioxidant power concentration. The resulting reduction in the pool of granulosa cells could impair reproductive function since this decrease could determine a disruption of progesterone and estradiol production, essential for the correct progression of the ovarian cycle [72].

A reduction in the number of mouse oocytes and granulosa cells by an apoptotic autophagy-related process associated with exposure to high PM2.5 was recently observed by Liao et al. [73] in the mouse model. Our results agree well with their hypothesis about PM2.5 adverse effects on female fertility by disrupting embryo development and quality. Also, Zhang et al. [16] showed that the in vitro treatment of granulosa cells with PM2.5 determined activity defect and apoptosis in a concentration-dependent manner.

The growth and differentiation of reproductive tissues, as well as the maintenance of fertility, depend on sex steroids. As for the female reproductive system, progesterone is essential in the post ovulation process of implantation as well as in the maintenance of pregnancy [74]. The results of our present experiments show that granulosa cells exposed to PM caused an inhibition of progesterone production for all the doses used. This reduction could possibly be attributable to the endocrine disruption exerted by the substances likely present in PM, as described above. It has been documented that human trophoblast cells exposed to PM2.5 produce lower levels of progesterone [75]. It is thought that PM can directly inhibit the phosphorylation state of PKA in JEG-3 (human choriocarcinoma cells) with a consequent decreased expression of proteins involved in progesterone production; this event seriously impairs reproductive function, considering the key role of this hormone in several processes such as pregnancy maintenance. Pillai et al. [76] and Nampoothiri et al. [77] have documented that exposure of animal models to heavy metals, such as Pb and Cd alone or in combination, determines a reduction of estradiol and progesterone serum levels, possibly resulting from the decreased activity of steroidogenic enzymes.

PM also reduced the production of 17β-estradiol in granulosa cells at all concentrations considered. It is well known that the hormone 17β-estradiol plays a crucial role in regulating the ovarian cycle, influencing the follicular, ovulatory, and luteal phases. In addition, it is essential for maintaining female sexual characteristics.

It is worth noting that the reduction of ovarian steroid levels in animals treated with heavy metals is linked to a reduction in the number of developing follicles and granulosa cells [76,77]. Studies conducted on E2-deficient and aromatase knockout (ArKO) mice demonstrate a reduced number of primordial and primary follicles. Furthermore, it was shown that the decrease in follicle number could not be corrected by postnatal treatment with E2, demonstrating that the lack of this hormone irreversibly affects the early phases of folliculogenesis [78]. It has been shown that diesel exhaust contains substances such as PAHs or heavy metals (Cu, Pb, Zn, etc.) that display estrogenic, antiestrogenic, and antiandrogenic activity, therefore affecting gonadal steroidogenesis and gametogenesis [79]. Dang et al. [80] reported that exposing rats to PM2.5 resulted in a reduction in the expression of estrogen receptors α in uterine tissue with a dose-effect relationship. An epidemiological study reported a decrease of mean E2 levels in female urban traffic police officers during the follicular and luteal phases. On these experimental bases, exposure to PM could be the cause of altered plasma E2 concentration [81].

Nitric oxide plays a crucial role in the control of the ovarian cycle and reproductive processes. Granulosa cells exposed to PM showed an increase in the production of NO, an RNS (nitrogen free radical). Currently, there are no data in the literature analyzing the effect of PM on NO production in granulosa cells cultured in vitro. Nam et al. [82] reported that RNS, produced by PM2.5, could activate the protein KF-kB (nuclear factor kappa-light-chain-enhancer of activated B cells), involved in the production of molecules that fuel the inflammatory process. They also documented that NF-kB could activate NO through iNOS; these two events can significantly intensify downstream reactions, establishing a vicious circle mediated by inflammatory cells and cytokines that could compromise granulosa cell function. It has been experimentally shown that NO negatively regulates the apoptotic process in ovarian cells and inhibits the production of estradiol 17β and progesterone by granulosa cells [83]. NO is essential in the ovulatory process, since the expression of the enzymes eNOS and iNOS is upregulated by gonadotropins, and the ovulation is blocked by NOS inhibitors. iNOS is expressed in luteal cells, but its activity decreases with the development of the corpus luteum; subsequently, during luteolysis, NO up-regulates the synthesis of PGF-2α, determining the reduction of progesterone synthesis. It follows that an increase in NO caused by PM could lead to an imbalance in the production of this hormone, with consequences on the correct ovarian cycle.

As for corpus luteum endothelial cells, a reduction in NO production was observed with PM treatments at 5 and 10 µg/mL, while a significant increase was reported at the higher PM concentration. Weldy et al. [84] have demonstrated that, by exposing a lymph node endothelial cell line to diesel exhaust particles, an upregulation of the expression of eNOS and iNOS is observed and, consequently, this leads to an increase in NO production. It should be noted that NO displays angiogenic effects that could be linked to the process of tumor invasion [85]. This influence is crucial since angiogenesis represents the “conditio sine qua non” for a correct progression of the ovarian cycle, given that the ovarian follicle requires a complex vascular system to correctly reach maturation [55]. Angiogenesis plays a fundamental role in ovarian cells by providing oxygen, nutrients, and support for ovulation, follicular maturation, and tissue repair. An adequate balance of angiogenesis in these cells is essential for proper ovarian and reproductive function. Angiogenic activity, assessed by VEGF levels, was significantly stimulated by PM treatments in corpus luteum endothelial cells. Xu et al. [86] documented that exposure of mice to diesel exhaust particles promoted the expression of VEGF and HIF-1α in vitro, suggesting that these substances can induce angiogenesis through the hypoxia/HIF pathway. VEGF represents the key player of physiological and pathological angiogenesis in many tissues, including the ovary [87]. In addition, it has been shown that granulosa cell differentiation is modulated by the autocrine action of VEGF: in less differentiated granulosa cells derived from small follicles, this growth factor reduces proliferation and stimulates E2 production, while in large ones the effects are reversed: proliferation is increased, and E2 production is reduced. Therefore, VEGF could promote cell differentiation even in the early stages of antral follicle development [88]. In the corpus luteum, intense angiogenesis occurs; this dense capillary network allows endocrine cells to obtain oxygen, nutrients, and hormonal precursors essential for synthesizing and releasing large quantities of progesterone, which is essential for maintaining pregnancy. By disrupting normal angiogenesis in the corpus luteum, PM could compromise corpus luteum regression as well as the physiological release of progesterone.

Thus, based on these in vitro results we can hypothesize that disruption of the physiological NO and VEGF concentration can impair ovarian angiogenesis and consequently the maturation of ovarian follicles as well as the maintenance of an adequate ovarian function.

In general, data collected in this study expand the knowledge, currently very scarce or often absent, on the effects of PM on reproductive cells. The environmental presence of these contaminants could affect the reproductive fitness of animals and humans.

## 5. Conclusions

Among the various pollutants that human activity emits into the atmosphere, microparticulate matter (PM) is of particular concern, to the point that it is indicated by the European Environmental Agency and the World Health Organization (WHO) as one of the most dangerous. The reproductive process is one of the main biological functions to be affected, since it is extremely sensitive to alterations of the internal as well as the external environment. Present research demonstrates that PM can impair the functions of the swine ovarian cell model. These data show alterations in ROS levels and in the activity of cellular antioxidant systems. Moreover, progesterone and 17β-estradiol concentrations, as well as VEGF levels and the accumulation of autophagic vacuoles, appear affected. Further studies are required to unravel the molecular mechanisms involved. However, the results are valuable to define criticisms in reproductive performances of sows present in farms located near industrial areas, high vehicular traffic, or waste disposal incinerators. These results raise concerns and suggest the need for a more in-depth study aiming to verify in vivo PM effects on the reproductive function of female mammals.

## Data Availability

The original contributions presented in this study are included in the article. Further inquiries can be directed to the corresponding author.
